# Tuberculosis Management Practices by Private Practitioners in Andhra Pradesh, India

**DOI:** 10.1371/journal.pone.0071119

**Published:** 2013-08-13

**Authors:** Shanta Achanta, Jyoti Jaju, Ajay M. V. Kumar, Sharath Burugina Nagaraja, Srinivas Rao Motta Shamrao, Sasidhar Kumar Bandi, Ashok Kumar, Srinath Satyanarayana, Anthony David Harries, Sreenivas Achutan Nair, Puneet K. Dewan

**Affiliations:** 1 World Health Organization (WHO) Country Office in India, New Delhi, India; 2 Central TB Division, Directorate General of Health Services, Ministry of Health and Family Welfare, Government of India, New Delhi, India; 3 State TB Cell, Directorate General of Health Services, Ministry of Health and Family Welfare, Government of Andhra Pradesh, Hyderabad, India; 4 District TB Centre, Directorate General of Health Services, Ministry of Health and Family Welfare, Government of Andhra Pradesh, Visakhapatnam, India; 5 International Union Against Tuberculosis and Lung Disease (The Union), South-East Asia Regional Office, New Delhi, India; 6 International Union Against Tuberculosis and Lung Diseases (The Union), Paris, France; McGill University, Canada

## Abstract

**Setting:**

Private medical practitioners in Visakhapatnam district, Andhra Pradesh, India.

**Objectives:**

To evaluate self-reported TB diagnostic and treatment practices amongst private medical practitioners against benchmark practices articulated in the International Standards of Tuberculosis Care (ISTC), and factors associated with compliance with ISTC.

**Design:**

Cross- sectional survey using semi-structured interviews.

**Results:**

Of 296 randomly selected private practitioners, 201 (68%) were assessed for compliance to ISTC diagnostic and treatment standards in TB management. Only 11 (6%) followed a combination of 6 diagnostic standards together and only 1 followed a combination of all seven treatment standards together. There were 28 (14%) private practitioners who complied with a combination of three core ISTC (cough for tuberculosis suspects, sputum smear examination and use of standardized treatment). Higher ISTC compliance was associated with caring for more than 20 TB patients annually, prior sensitization to TB control guidelines, and practice of alternate systems of medicine.

**Conclusion:**

Few private practitioners in Visakhapatnam, India reported TB diagnostic and treatment practices that met ISTC. Better engagement of the private sector is urgently required to improve TB management practices and to prevent diagnostic delay and drug resistance.

## Introduction

As per the World Health Organization (WHO) global TB control report 2011, India continues to bear the highest global burden of TB with an estimated 2.3 million incident cases per annum accounting for more than one-fourth of global TB incidence [1]. The Revised National TB Control Programme (RNTCP) is being implemented in the country since 1997 with complete country coverage by March 2006, with TB diagnostic and treatment services integrated throughout the public health infrastructure. Despite this success, India has a very large unregulated private medical sector. Although data are sparse, there could be as many TB patients seeking health care in private sector as there are in the public [2]–[5]. A recent analysis of drug sales in the country circumstantially supported this interpretation [6]. Effective engagement of the private sector is vital to facilitate national TB control strategies.

There have been significant efforts to engage the private sector with the RNTCP through various Non-Governmental Organization/Private Provider (NGO/PP) schemes [7]. The Indian Medical Association (IMA), the country’s largest representative voluntary organization of qualified medical practitioners of modern medicine, is an important partner of the RNTCP and has adopted the International Standards for TB Care (ISTC). These standards describe a widely accepted level of TB care that all health care practitioners, both public and private, should seek to achieve, and the essential public health responsibilities that they must carry out [8]. The ISTC includes 21 standards that cover four broad areas: diagnosis, treatment, addressing HIV and other co-morbid conditions and public health responsibilities. Of the 21 standards, six describe diagnostic and seven describe treatment standards.

Many studies from India have documented that the private sector often deviates from the standard, internationally recommended, TB management practices [9]–[12]. Inappropriate diagnostic practices may lead to diagnostic delay, perpetuating TB transmission. Inappropriate treatment practices risk amplification of drug resistance.

Very few prior studies on private TB management practices have included representative samples of providers or assessed risk factors for such practices. We conducted a representative survey of formally qualified private medical providers (PMP) in one district of Andhra Pradesh, South India to evaluate self-reported TB diagnostic and treatment practices against the benchmark practices articulated in the ISTC. We further evaluated factors associated with compliance with ISTC-recommended practices.

**Figure 1:**
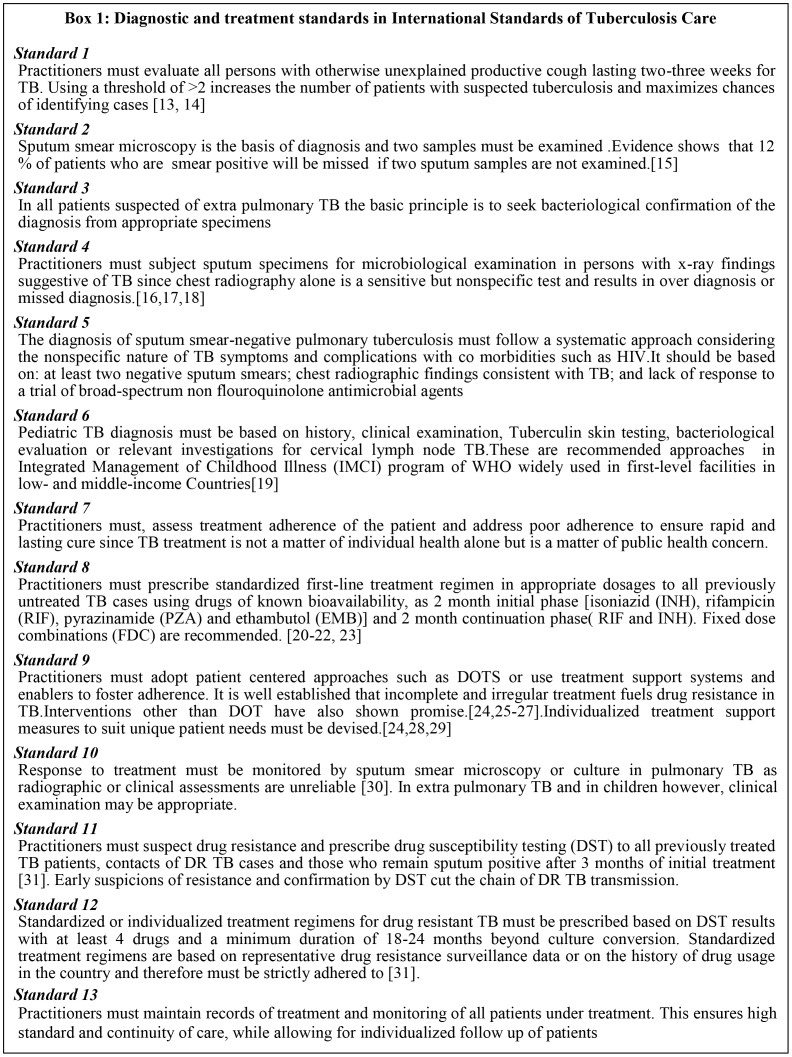
Box 1- Diagnostic and treatment standards in International Standards of Tuberculosis Care. The principles of diagnostic and treatment standards in the International Standards of TB Care with their rationale and references are summarized.

The principles of diagnostic and treatment standards in the ISTC with their rationale [13]–[31] are summarized in Box 1 (Figure 1).

## Methods

### Ethics Approval

Ethics approval was obtained from Ethics Advisory Group of the International Union against Tuberculosis and Lung Disease (The Union) and the ethics committee of the National Tuberculosis Institute, Bangalore. Administrative approvals were obtained from the State authorities for conducting this study. A written informed consent was taken from each participant and confidentiality was assured as data collection formats were maintained securely by programme staff and electronic databases contained no personal identifiers.

### Study Design

This was a cross sectional survey of Private Medical Practitioners (PMP) in Visakhapatnam district, Andhra Pradesh, South India.

### Setting

The district of Visakhapatnam (population 4.6 million) is located in the State of Andhra Pradesh India with a total population. of ∼83 million and 23 administrative districts. The district has been consistently achieving the RNTCP objectives with cure rate of more than 90% and new smear positive case detection rate of more than 80%. Nearly 60% of population is urban and 40% is rural of which 15% lives in tribal areas. This district was purposefully chosen because of the large and diverse target population of PMP’s (∼4000), with ∼70% practicing in the urban areas and the long history of efforts to sensitize private practitioners. The RNTCP was implemented in 1997 and on a regular basis over the years, district and state health authorities have conducted outreach and sensitization on TB for private practitioners. In addition, since 2008 a Global Fund supported project–implemented by the Indian Medical Association–has conducted systematic sensitization and training of all qualified allopathic providers about the RNTCP.

### Study Population and Study Period

The study was conducted during December 2010 to July 2011.The study population included all PMPs with a medical degree from any recognized system of medicine (Modern medicine (allopathy), Ayurveda, Unani, Siddha and Homeopathy). All PMPs with private practice irrespective of their association with the government health services were included in the study. The ‘master list’ of PMPs was generated from a line list of enrolled doctors from the IMA directory, the list of all PMPs maintained by the programme and a list of practitioners from the Association of AYUSH (Ayurveda, Unani, Siddha, and Homeopathy). This master list was reviewed for duplicates and cleaned of those practitioners who might not be in current practice (i.e. died, moved out of district or were not in practice currently).

### Sample Size and Sampling

The sample size was calculated as 296 based on the assumptions of 20% expected frequency of ISTC adherence, with 5% precision and 20% non-response among eligible participants. The required number of PMPs was selected by systematic random sampling. Prior appointment with each PMP was confirmed over the phone, a participant information sheet was handed over to the PMP and written consent for her/his participation was obtained before the interview. If the practitioner was not available during the first visit, at least two subsequent visits were made at intervals of one week within a month. If a selected PMP was not available for interview or a non-responder, the next consecutive PMP from the master list was included in the sample.

### Data Variables

Data collected include the system of medicine practiced by the PMPs, duration of practice, the type of private practice (exclusive private practice or combined with public practice), average number of TB patients seen in a month and history of exposure to RNTCP sensitization. The key outcome variables were the number (proportion) of practitioners following the ISTC Standards of Diagnosis (Standards 1 to 6) and Treatment (Standards 7 to 13).

### Data Collection

**Figure 2:**
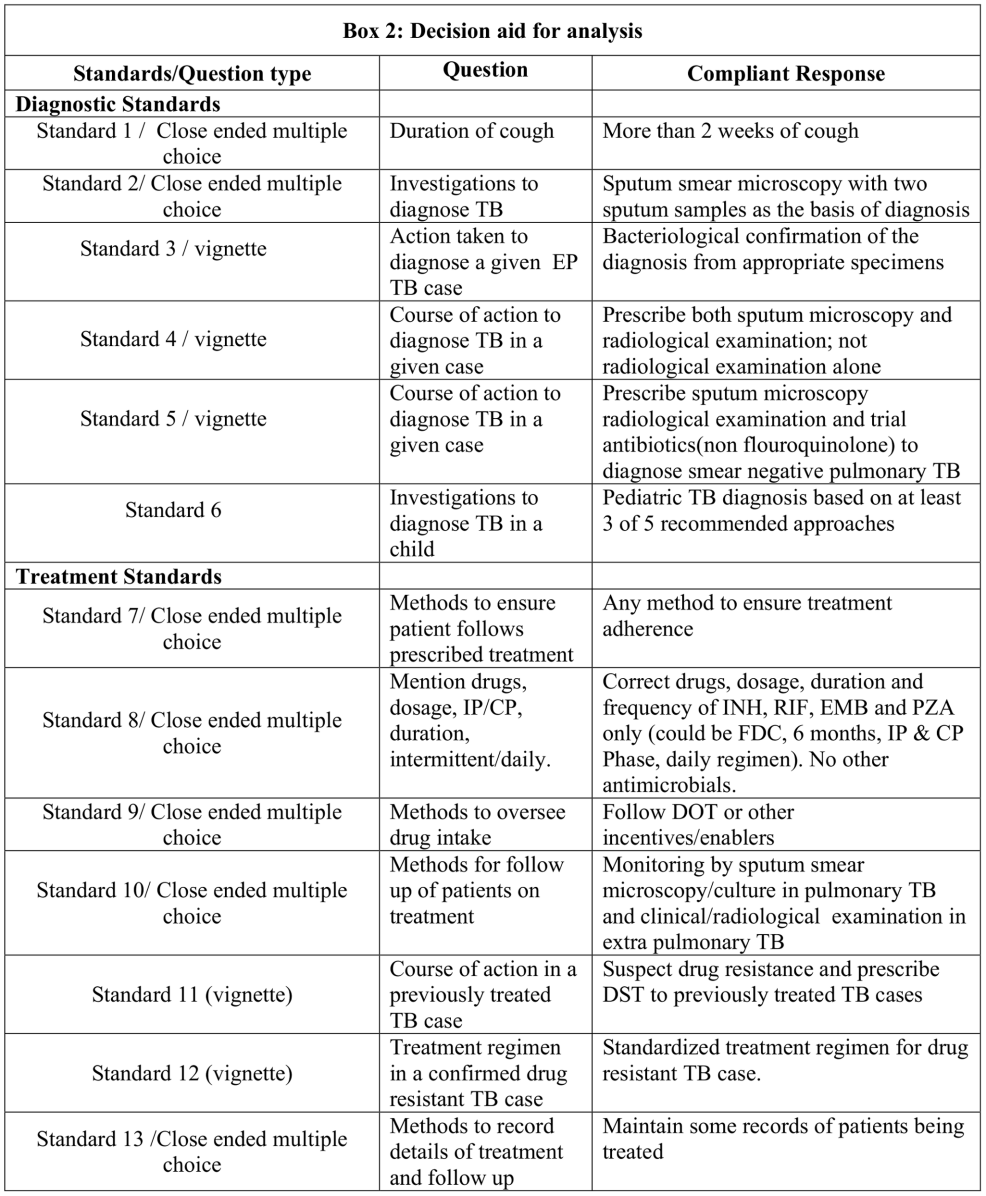
Box 2- Decision aid for analysis. This is a decision aid for the purpose of analysis of data; the type of question asked in the questionnaire, subject of the question and the responses which should be considered as compliant against each diagnostic and treatment standard are summarized.

The semi-structured questionnaire was designed and pilot tested on ten providers outside the sample population and then the standardized questionnaire was developed. The questionnaire consisted of vignettes and multiple choice close ended questions. The vignettes presented a case history (narrative) to the practitioner, who then provided his/her responses on diagnostic and treatment choices as the case may be. If the answer met the expected standard response, it was rated to be correct and if it deviated from the principles in the standards it was rated as incorrect. Questions for Standards 3, 4, 5 for evaluation of diagnostic practices and questions for standards 11, 12 for evaluation of treatment practices were vignette based. A decision aid was prepared to assist in clearly sorting out ‘compliant’ and ‘non compliant’ responses to the questions as summarized in Box 2 (Figure 2).

Eight medical officers working for the RNTCP were trained by the investigators to impart interview skills and administer the semi-structured questionnaire through personal interviews. These interviewers underwent robust training at the district TB centre for one day and it was ensured that the interviewers were well conversant with all the topics and practiced at least three interviews on non-study participants. Each interviewer was assigned a geographical area with a definite number of private practitioners to be interviewed.

### Data Quality Assurance

In order to elicit optimum responses and minimize interviewer bias, the interviewers were medical practitioners who were not from the same location or related to the sampled private practitioners. Interviewers scrutinized each other’s completed questionnaire before data entry. All the data collection forms were checked for completeness and consistency by the principal investigator and corrections were made.

### Data Handling and Analysis

Double data entry was done in a pre-designed format of Epi Info Version 3.5.1 independently by two data entry operators. Both the data bases were compared and discrepancies were resolved by referring to the original data collection formats. PMPs were classified into those following ISTC or not based on whether they followed three selected and core ISTC standards (use of cough for 2 weeks for identifying Pulmonary TB suspects; sputum microscopy for diagnosing Pulmonary TB; use of the 6–8 months fixed dose combination (FDC) regimen for treating TB). Any PMP adhering to all three standards was classified as “compliant with ISTC” while those only adhering to 2 or less were classified as “non-compliant”. All questions in the questionnaire were not mandatory considering the varied nature of practices among the PMPs. There was an option of no response in the questionnaire. For the purpose of our analysis a PMP was considered for analysis only for the standards he responded to. All analysis was done using Epi Info analysis software. Proportions were compared using frequency distributions and the chi-squared test. Relative risks and 95% confidence intervals were derived, and a ‘p’ value of less than or equal to 0.05 was considered as statistically significant.

## Results

**Figure 3:**
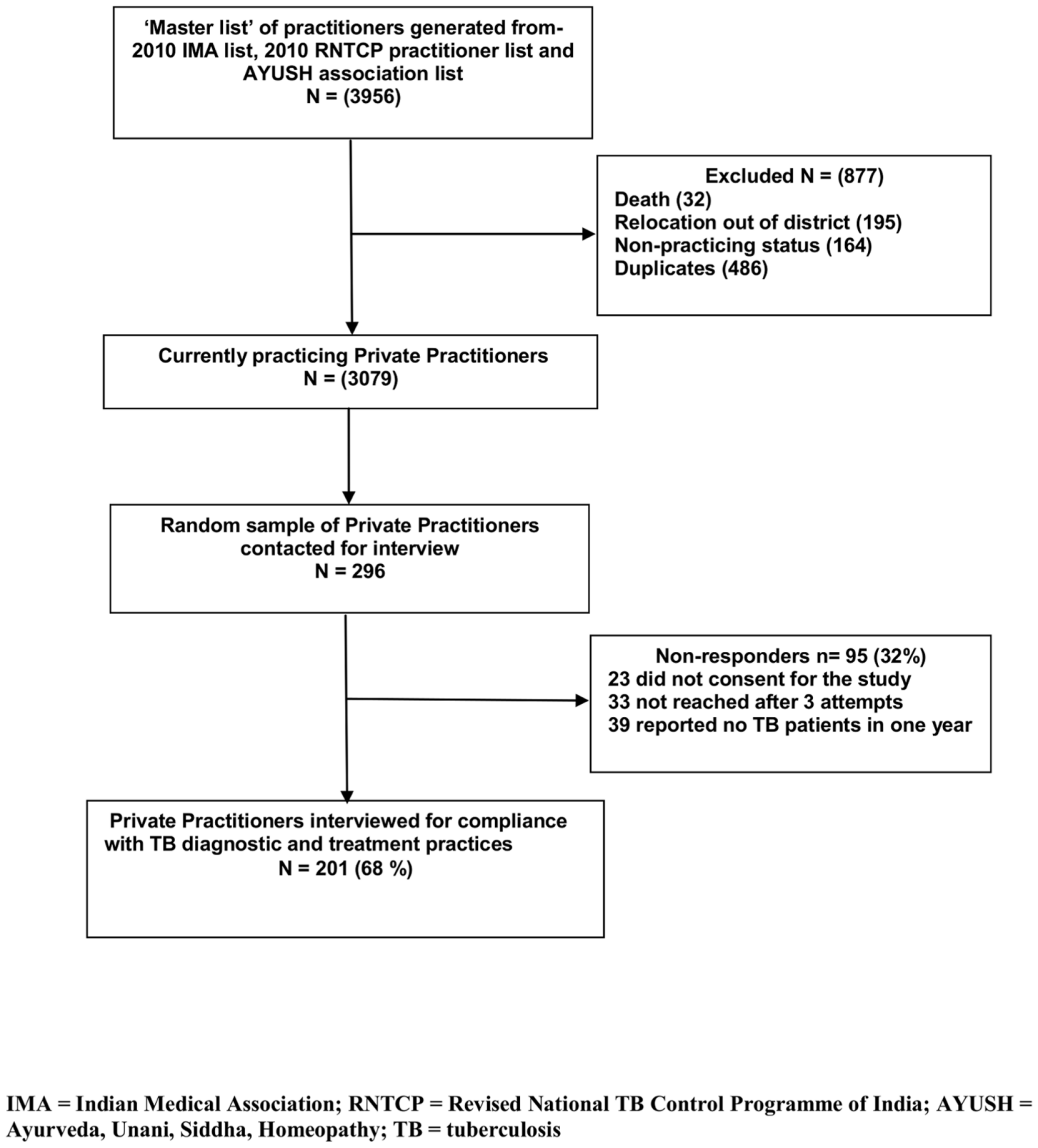
Selection of Private Practitioners (study participants) in Visakhapatnam, Andhra Pradesh. A master list of 3956 private practitioners was prepared from the year 2010 list of IMA*, RNTCP*** practitioners and AYUSH**. Of these 877 were excluded due to death, 195 relocated out of district, 164 non-practicing status and 486 were duplicate entries. Of the remaining 3079 practitioners, a random sample of 296 were contacted for interview. Of those contacted, 95 (32%) were non-responders, 23 did not consent for the study, 33 not reached after 3 attempts and 39 reported seeing zero TB**** patients in one year. So, Private Practitioners interviewed for compliance with TB diagnostic and treatment practices were N = 201 (68%). IMA* = Indian Medical Association; RNTCP** = Revised National TB Control Programme of India; AYUSH*** = Ayurveda, Unani, Siddha, Homeopathy; TB**** = tuberculosis.

Of a total number of 3,956 PMPs in the district, 296 were randomly selected for the study, of whom 201 (68%) were assessed for diagnostic and treatment practices against ISTC (**Figure 3**). The mean age of PMPs was 35 years and 154 (75%) were male. Other characteristics of the PMPs were as follows: 84% practiced allopathic medicine and had a bachelor’s degree (MBBS); 82% had been in medical practice for 10 years or longer, with the majority solely in private practice; 81% had treated 20 or fewer TB patients in the previous year, and 43% had been sensitized in RNTCP guidelines. There were 18 PMPs from the Medical colleges. All of them were sensitized in RNTCP; 12 of them were compliant with Standard 1, 10 with Standard 2, 7 followed Standard 8 and only 4 followed all the three standards together (not shown in the table ).

Characteristics of responders and non-responders are compared in **Table 1**. Non responders were similar to responders by age, sex and qualification.

**Table 1:** Characteristics of responders and non-responders among private practitioners in Visakhapatnam, Andhra Pradesh.

Characteristics	Responders N (%)	Non responders N (%)	Total	P value
**Total**	**201**	**95**	**296**	
**Age (years)**				
20–40	65 (32.3)	28 (29.4)	93	
41–60	91 (45.2)	47 (49.4)	138	0.53
>61	45 (22.4)	20 (21.1)	65	0.92
**Gender**				
Male	154 (76.6)	69 (72.6)	223	
Female	47 (23.4)	26 (27.4)	73	0.46
**Total**	**179**	**95**	**274**	
**Qualification**				
AYUSH	27 (13.4)	11 (11.6)	38	
Modern medicine	152 (86.6)	84 (88.4)	236	0.43

**Responders = those who were administered the questionnaire, non responders = those who were not administered the questionnaire, AYUSH = Ayurveda, Unani, Siddha, Homeopathy, P value ≤0.05 = significant.**

The number and proportion of PMPs following ISTC standards for diagnosis are shown in **Table 2** **.** The number of responders considered for analysis against each standard changes based on whether or not a response was recorded against that question. Two thirds of PMPs followed standards for suspecting pulmonary TB based on a cough of 2 weeks or more but fewer than 45% adopted sputum smear examination as the standard diagnostic practice. About 50% of PMPs followed the other ISTC diagnostic standards, with the exception of pediatric diagnostic approaches where the proportion was higher at 77%. Only 11 (6%) of PMPs followed all 6 diagnostic standards together.

**Table 2:** Private Practitioners adhering to ISTC* diagnostic practices in Visakhapatnam, Andhra Pradesh.

Diagnostic Practices	Total number respondingto the question	Number (%) adhering toISTC standards
**Standard 1**	201	137 (68)
Cough of 2–3 weeks to suspect TB		
**Standard 2**	198	87 (44)
Two sample Sputum smear examination for diagnosis of Pulmonary TB		
**Standard 3**	176	93 (53)
Extra Pulmonary TB diagnosis based on appropriate investigations		
**Standard 4**	182	85 (47)
Sputum microbiological examination in those with radiological findings suggestive of TB		
**Standard 5**	155	75 (48)
Diagnose sputum smear-negative pulmonary TB based on both sputum microscopy & X-ray		
Use of the right trial antibiotic*	161	85 (53)
**Standard 6**	188	144 (77)
Diagnose Pediatric TB based on at least 3 of 5 recommended approaches **		
**Standard 1 and 2 together**	201	68 (34)
**Standard 3 and 4 together**	192	42 (22)
**Standard 5 and 6 together**	197	56 (28)
**Standards 1 to 6 altogether**	201	11 (6)

*(International Standards of TB Care).

**TB = tuberculosis;**

*Response to what antibiotic was used as trial antibiotic. 85 (53%) used non-fluoroquinolones.

**5 approaches included history, clinical examination, Tuberculin skin testing, bacteriological evaluation or relevant investigations to diagnose e.g. cervical lymph node TB.

The number and proportion of PMPs following ISTC standards for treatment are shown in **Table 3**. Less than 40% of PMPs prescribed standard TB treatment regimens, practiced directly observed treatment (DOT), prescribed culture and drug susceptibility testing or appropriate treatment for drug-resistant TB. Adoption of methods to ensure treatment adherence and the follow up of cases with sputum smear microscopy was higher at about 85%. Only 27% maintained patient clinical records. Only one PMP followed all 7 treatment standards altogether.

**Table 3:** Private Practitioners adhering to ISTC^*^ treatment practices in Visakhapatnam, Andhra Pradesh.

Treatment Practices	Total number respondingto the question	Number (%) adhering toISTC standards
**Standard 7**	188	159 (85)
Adopt methods to ensure adherence to treatment		
**Standard 8**	177	61 (35)
Prescribe standard TB treatment regimen 2HRZE and 4RH (daily and thrice weekly) in FDC		
**Standard 9**	195	40 (21)
Practice DOT or foster adherence with treatment supporter or other enablers		
**Standard 10**	187	160 (85)
Follow up cases with sputum microscopy		
**Standard 11**	151	59 (39)
Prescribe culture and drug susceptibility testing for previously treated TB cases		
**Standard 12**	97	37 (38)
For diagnosed DR TB, prescribe standardized regimen, or individualized TB treatment regimen based on availability of drug susceptibility testing		
Standard 13	165	44 (27)
**Maintain the patient clinical record**		
**Standard 7 and 8**	191	52 (27)
**Standard 9 and 10**	201	16 (8)
**Standard 11 and 12**	200	23 (12)
**All treatment standards (7–13)**	201	1 (1)

*(International Standards of TB Care). H = INH, R = Rifampicin, Z = Pyrazinamide, E = Ethambutol, FDC = Fixed Drug Combinations, DOT- Directly observed treatment; TB = Tuberculosis; DR TB = Drug resistant TB.

There were 28 (14%) PMPs who were compliant with the 3 selected core ISTC standards. Characteristics related to compliance with each of these standards and all 3 ISTC standards together are shown in **Table 4**. Higher levels of compliance with all three standards were observed among those practitioners practicing AYUSH, those who saw more than 20 TB patients in the previous year and those who were sensitized to RNTCP guidelines.

**Table 4:** Characteristics of private practitioners in relation to compliance with 3 selected core ISTC^*^ in Visakhapatnam, AP.

	Standard 1	Standard 2	Standard 8	Standard 1+2+8	
Characteristic	N (% )	N (% )	N (% )	N (% )	RR (95% CI)
**Gender:**					
Male (n = 154)	106 (70)	67(45)	44 (32)	23 (15)	1.2 (0.5–3.1)
Female(n = 47)	24 (57)	17(41)	16 (47)	5 (12)	
**Qualification:**					
AYUSH (n = 27)	22 (82)	18 (69)	9 (35)	8 (30)	2.4 (1.2–4.9)
Modern Medicine (n = 152)	96 (63)	63 (42)	47 (35)	19 (13)	
**Years in Practice:**					
Less than 10 (n = 32)	22 (69)	20 (65)	13 (46)	7 (22)	1.9 (0.8–4.3)
10 or more (n = 153)	102 (67)	56 (37)	44 (33)	17 (11)	
**Type of Practice:**					
Private and Government (n = 71)	46 (65)	35 (50)	27 (44)	14 (20)	1.7 (0.8–3.7)
Private only (n = 100)	68 (68)	37 (38)	29 (32)	11 (11)	
**TB patients seen last year:**					
21 and above (n = 53)	40 (76)	31 (56)	21 (45)	12 (22)	2.1 (1.1–4.1)
20 or less (n = 148)	97 (66)	56 (39)	40 (30)	16 (11)	
**Sensitized to RNTCP guidelines:**					
Sensitized (n = 87)	67 (77)	53 (61)	36 (43)	20 (23)	3.2 (1.5–7.0)
Not sensitized (n = 113)	69 (61)	34 (31)	25 (27)	8 (7)	

*(International Standards of TB Care).

**Core ISTC Standards: Standard 1 = Using 2–3 weeks cough for identification of Pulmonary TB suspects; Standard 2 = Using sputum smear microscopy examination; and Standard 6 = Treatment with the standard 6-month regimen; TB = Tuberculosis; AYUSH = Ayurveda, Unani, Siddha, Homeopathy; RNTCP = Revised National TB Control Programme; RR = Relative Risk has been calculated for standard 1+2+8; CI = Confidence Interval.**

## Discussion

This is the first study in India on a representative sample of qualified private practitioners which has evaluated diagnostic and treatment practices for tuberculosis, relative to international standards of care. Previous studies on PMPs have been conducted in India [4], [9]–[12] but none of them have assessed adherence to ISTC-recommended professional practices. Compliance to ISTC standards was quite poor overall; only 6% of PMPs followed all 6 diagnostic standards together and only <1% of PMP followed all seven treatment standards together. Even using just the core three standards (i.e. cough for PTB suspects, referral for sputum smear microscopy examination, and treatment with the standard 6-month regimen) less than 15% of PMPs followed the three standards together. These findings suggest that the TB diagnosis and treatment practices uncommonly reach even the most basic standards of TB care.

Our data provided several findings that warrant closer examination. First, doctors with qualification from alternate systems of medicine (i.e. AYUSH doctors) surprisingly performed better than those with basic medical and specialist degrees. We speculate that diagnostic and treatment standards may be more important to providers with less extensive training in tuberculosis, hence adherence was higher. This group constituted 15% of the sample of providers in our survey. Existing interventions to engage the private sector such as the IMA-RNTCP project, systematically neglect AYUSH practitioners; our data thus suggest that AYUSH providers are important.

Second, compliance to the two important standards of diagnosis was only 34%, meaning only few rightly suspected TB and prescribed sputum microscopy for diagnosis of TB as their first choice. Only 47% diagnosed TB based on both sputum microbiological examination and X-ray. This indicates the over reliance on other investigations like ESR and other serological tests in the private sector [32]. It could be due to the tendency of practitioners to adopt investigations that give immediate results and pressure on them to provide faster results to their clients. Vigorous efforts should be made by the district to disseminate information to the practitioners on the availability of newer standard diagnostics to help change their prescribing practices [33], [34]. Strategies adopted by private drug or diagnostic companies to influence and market their products like regular visits and attractive incentives to supporting practitioners may work well for the programme and may be piloted in a few districts. Private labs that already offer these facilities can be identified, accredited and encouraged to participate.

Third, it was noted that the PMPs addressed poor adherence to treatment by methods like ‘intensive counseling’, ‘follow up by phone calls’ and also ‘periodic check ups’. However, in response to question addressing standard 9, very few observed any means of supervised treatment. This is encouraging compared to earlier studies [9] and is a potential area to introduce newer methods of ensuring adherence. Studies have shown that attitude of health care staff is very important in determining treatment adherence [35] and most PMPs would be looking for means to build rapport with their patients. Directly Observed Treatment (DOT) can be advocated as the preferred method; however PMPs who can document and report treatment adherence by other means should also be supported.

Fourth, only one-third prescribed the correct treatment regimen for a new case of Tuberculosis with no previous history of TB treatment. Two third prescribed a large variety of different prescriptions in terms of drugs, dosage and duration. Particularly disturbing was the finding that more than 50% prescribed second line anti TB drugs like fluoroquinolones, as trial antibiotics to initial smear negative cases, a practice specifically recommended against in ISTC and any TB treatment guidelines. These findings are reminiscent of a recent report from Mumbai where a small convenience sample of practitioners reported use of more than 80 different prescriptions [36]. Poor TB treatment practices are well known to increase the risk of the development of anti-TB drug resistance. The high prevalence of PMPs reporting treatment practices not aligned with the standards of TB treatment warrants considerable alarm.

Very few PMPs maintained any record of the patients they treated consistent with findings from earlier studies [37]. Those that responded favorably could only show some documents recording the date of starting treatment and the follow up investigations advised. Programme must devise easy methods to encourage reporting and recording of these cases. This will provide an estimate of how many cases are actually treated under the private sector.

Practitioners vary in their levels of competence and this also varies between practitioners from public and private sector [38], [39]. Measure of competency is a function of their compliance to technical as well as quality of care standards. Evidence from literature reveals that PMPs compared to public sector exhibit serious lapses in complying with prescribed technical standards of health care [40].On the other hand, there is evidence that elements of quality of care such as geographical accessibility, shorter waiting periods, flexible hours, confidentiality, availability of staff, belief in values of relationships are reasons why patients seek health care from PMPs in preference to public sector [41]. Both technical standards and quality of care aspects are critical for TB control. This study prompts further research to study quality of care issues among PMPs in management of TB cases.

### Limitations of the Study

In this cross sectional, questionnaire-based study, only 68% of selected candidates were interviewed and hence a response bias could have been introduced. However, comparison of baseline characteristics among non-responders and responders shows no difference in the two groups by age, sex and qualification. This is reassuring and suggests that effect of response bias on the overall results might have been minimal. Our sampling frame included only qualified providers and hence the findings cannot be generalized to the vast number of non-qualified health care providers in the district. Most importantly, data on TB diagnostic and treatment practices were as reported by the PMPs and may not be exactly the same as what they actually practice. Assessments in quality of care should seek to include empiric data on actual treatment practices, which may differ in important ways from reported practices. Furthermore, other studies have suggested that TB patients shop for effective treatment and sometimes later shift to government services, so the final impact of these diagnostic and treatment practices on patient outcomes is unknown but is beyond the scope of this study.

### Conclusion

Our study found that compliance to internationally recommended standards of TB diagnosis and treatment was uncommon among the PMPs in Visakhapatnam, India. As India plans towards universal access to TB care in the next five year plan (2012–17), programme needs to prioritize the involvement of Private sector to its fullest with unique and innovative strategies.
